# Effect of *Xanthium Strumarium* on HIV-1 5′-LTR Transcriptional Activity and Viral Reactivation in Latently Infected Cells

**DOI:** 10.3389/fphar.2021.720821

**Published:** 2021-08-06

**Authors:** Chao-Jung Chen, Mu-Lin Chiu, Chien-Hui Hung, Wen-Miin Liang, Mao-Wang Ho, Ting-Hsu Lin, Xiang Liu, Hsinyi Tsang, Chiu-Chu Liao, Shao-Mei Huang, Yi-Fang Wu, Yang-Chang Wu, Te-Mao Li, Fuu-Jen Tsai, Ying-Ju Lin

**Affiliations:** ^1^ Genetic Center, Proteomics Core Laboratory, Department of Medical Research, China Medical University Hospital, Taichung, Taiwan; ^2^ Graduate Institute of Integrated Medicine, China Medical University, Taichung, Taiwan; ^3^ School of Chinese Medicine, China Medical University, Taichung, Taiwan; ^4^ Graduate Institute of Clinical Medical Sciences, Chang-Gung University, Taoyuan, Taiwan; ^5^ Division of Infectious Diseases, Chang Gung Memorial Hospital Chiayi Branch, Chiayi, Taiwan; ^6^ Department of Health Services Administration, China Medical University, Taichung, Taiwan; ^7^ Section of Infectious Diseases, Department of Internal Medicine, China Medical University Hospital, Taichung, Taiwan; ^8^ Department of Internal Medicine, School of Medicine, China Medical University, Taichung, Taiwan; ^9^ National Institute of Allergy and Infectious Diseases, National Institutes of Health, Bethesda, MD, United States; ^10^ Chinese Medicine Research and Development Center, China Medical University Hospital, Taichung, Taiwan; ^11^ Department of Biotechnology and Bioinformatics, Asia University, Taichung, Taiwan

**Keywords:** human immunodeficiency virus type 1 latency, Chinese herbal medicine, X. strumarium, 5′-long terminal repeat, nuclear regulatory proteins

## Abstract

Chinese herbal medicines (CHMs) are widely used in Asian countries. They show multiple pharmacological activities, including antiviral activities. The 5′-long terminal repeat (LTR) region of HIV-1, required for viral transcription, is a potential drug target for HIV-1 reactivation and intrinsic cell death induction of infected or latently infected cells. Modulation of HIV-1 reactivation requires interactions between host cell proteins and viral 5′-LTR elements. By evaluation of two CHMs- *Xanthium strumarium* and *Pueraria montana*, we found that 1) *X. strumarium* reactivated HIV-1 latently infected cells in J-Lat 8.4, J-Lat 9.2, U1, and ACH-2 cells *in vitro*; 2) 27 nuclear regulatory proteins were associated with HIV-1 5′-LTR using deoxyribonucleic acid affinity pull-down and LC-MS/MS analyses; and 3) among them, silencing of *XRCC6* reactivated HIV-1 5′-LTR transcriptional activity. We found that *X. strumarium* inhibits the 5′-LTR associated XRCC6 nuclear regulatory proteins, increases its viral 5′-LTR promoter transcriptional activity, and reactivates HIV-1 latently infected cells *in vitro*. These findings may contribute to understanding the 5′-LTR activity and the host cell nuclear regulatory protein machinery for reactivating HIV-1 and for future investigations to eradicate and cure HIV-1 infection.

## Introduction

Highly active antiretroviral therapy (ART) for human immunodeficiency virus type 1 (HIV-1) infection has dramatically reduced HIV-1 viral loads and decreased morbidity and mortality (Granich et al., 2010). However, HIV-1 patients cannot be cured due to the HIV-1 viral genome integrated into human genome and become long-lived memory T cells with provirus as HIV-1 reservoirs (Coiras et al., 2009; Richman et al., 2009). HIV-1 remains in a latent state within resting CD4^+^ T cells (one of the HIV-1 latently infected cells) even in the presence of HAART (Warren et al., 2020).

The HIV-1 retroviral genome contains a duplication of the viral transcriptional control sequences in the 5′**-** and 3′**-** long terminal repeat (LTR) regions (Klaver and Berkhout, 1994; Roebuck and Saifuddin, 1999). The viral 5′-LTR transcriptional activity is much higher than the 3′-LTR transcriptional activity (Klaver and Berkhout, 1994). Latency reversal agents (LRAs) can demethylate and induce HIV-1 5′-LTR transcriptional activity (Ishida et al., 2006). These studies have prompted studies on epigenetic and transcriptional regulation of the 5′-LTR as a potential drug target for HIV-1 reactivation and intrinsically induce cell death in infected cells (Baxter et al., 2018; Rao et al., 2021). Studies on LRAs that target the 5′-LTR have highlighted the potential for eradication and cure of HIV-1 infection (Chun et al., 1998; Chun et al., 1999; Kulkosky et al., 2001; Wang et al., 2005; Archin et al., 2009; Kauder et al., 2009). LRAs may provide a shock and kill strategy for eradicating HIV/AIDS by stimulating HIV-1 replication and transcription in HIV-1 latently infected cells.

In Taiwan, Chinese herbal medicines (CHMs) have been widely used in HIV-1 infected patients (Tsai et al., 2018). *Xanthium strumarium subsp. sibiricum* (*Patrin ex Widder*) *Greuter* (*X. strumarium*) (Family Asteraceae), also called *Cocklebur* or *Xanthii Fructus*, is a CHM. *X. strumarium* has multiple pharmacological activities including the antiviral activity (Guo et al., 2013; Lin et al., 2014; Shi et al., 2015; Amin et al., 2016; Jiang et al., 2017; Fan et al., 2019; Kim et al., 2019; Xia et al., 2020a; Xia et al., 2020b; Yuan, 2020). As a traditional Chinese herbal medicine, *Pueraria montana* var. *lobata* (*Willd.*) *Sanjappa and Pradeep* (*P. montana*) (Family Fabaceae) has antiviral activity against the human respiratory syncytial virus (HRSV) (Lin T.-J. et al., 2013). Furthermore, *P. montana* can effectively inhibit HIV-1 entry into cells by suppressing viral attachment to the cell surface (Mediouni et al., 2018). However, there are no studies on the role of *X. strumarium* and *P. montana* in eradicating HIV-1 viral reservoirs.

Evaluating the two CHMs- *X. strumarium* and *P. montana*, we found that *X. strumarium* reactivated HIV-1 latently infected cells *in vitro*. HIV-1 5′-LTR DNA affinity pull-down coupled with LC-MS/MS analyses identified 27 nuclear regulatory proteins in *X. strumarium*-treated cells. Characterization of the HIV-1 5′-LTR promoter activity by silencing these newly identified nuclear regulatory proteins was also examined. To our knowledge, this is the first study to describe *X. strumarium* exerting a HIV-1 reactivation activity via a mechanism that modulating the composition of nuclear regulatory proteins in the 5′-LTR region.

## Materials and Methods

### Cells

J-Lat cell lines (clones 8.4 and 9.2) were clonal Jurkat cells with a latent HIV-1 provirus, in which the enhanced green fluorescent protein (EGFP) replaces the *nef* coding sequence and a frameshift mutation in the *env* gene (Jordan et al., 2003). J-Lat cells were monitored for HIV-1 reactivation by Western blot analysis because EGFP protein expression levels were controlled by the HIV-1 5′- LTR.

ACH-2 (T cell-derived) and U1 (promonocyte-derived) cell lines were cells with full-length proviruses, without EGFP replacing coding sequence [ACH-2 cells: there is a point mutation in the Tat-responsive element (TAR) (Emiliani et al., 1996), and U1 cells. There are mutations in Tat (Emiliani et al., 1998)]. ACH-2 and U1 cells were monitored for HIV-1 reactivation by detecting HIV-1 gene products (e.g., HIV-1 p24 virus capsid protein). J-Lat cells, ACH-2 cells, and U1 cells were grown in RPMI 1640 medium with 10% fetal bovine serum (FBS) (Gibco), 100 U/mL penicillin (Gibco), 100 U/mL streptomycin (Gibco), and 2 mM l-glutamine (Gibco).

TZM-bl cells are clonal HeLa cells that stably express large cell surface receptors, including cluster of differentiation four receptors (CD4) and C-C chemokine receptor type 5 (CCR5) (Todd et al., 2012). Moreover, TZM-bl cells were cells with their genome integrated with copies of the firefly luciferase and beta-galactosidase genes under the HIV-1 5′-LTR control. TZM-bl cells were monitored for HIV-1 reactivation by detection of firefly luciferase activity or beta-galactosidase protein expression levels under the HIV-1 5′-LTR control. TZM-bl cells were grown in DMEM with 10% fetal bovine serum (FBS) (Gibco), 100 U/mL penicillin (Gibco), 100 U/mL streptomycin (Gibco), and 2 mM l-glutamine (Gibco). J-Lat, ACH-2, U1, and TZM-bl cells were obtained from the AIDS Research and Reference Reagent Program, National Institutes of Health (NIH), United States.

### Chinese Herbal Medicine and Related Marker Compounds

Crude herbal extract powders of the two Chinese herbal medicines (CHMs)- *Xanthium strumarium subsp. sibiricum* (*Patrin ex Widder*) *Greuter* (*X. strumarium*) (Family Asteraceae) and *Pueraria montana* var. *lobata* (*Willd.*) *Sanjappa and Pradeep* (*P. montana*) (Family Fabaceae) were provided by Chuang-Song-Zong Pharmaceutical Co., Ltd., one of the good manufacturing process (GMP)- pharmaceutical manufacturers for CHMs in Taiwan. These preparations have been described previously (Cheng et al., 2019). Fine and crude herbal extract powders were prepared by filtering through a 20-mesh metal sieve, then mixing 1.0 g of powder with 40 ml distilled water. After shaking overnight at 4°C, the mixture was filtered through a 100-mesh metal sieve. Filtrate (crude water extract) was sterilized using a 0.44 µm syringe filter and used for further evaluation.

**FIGURE 1:**
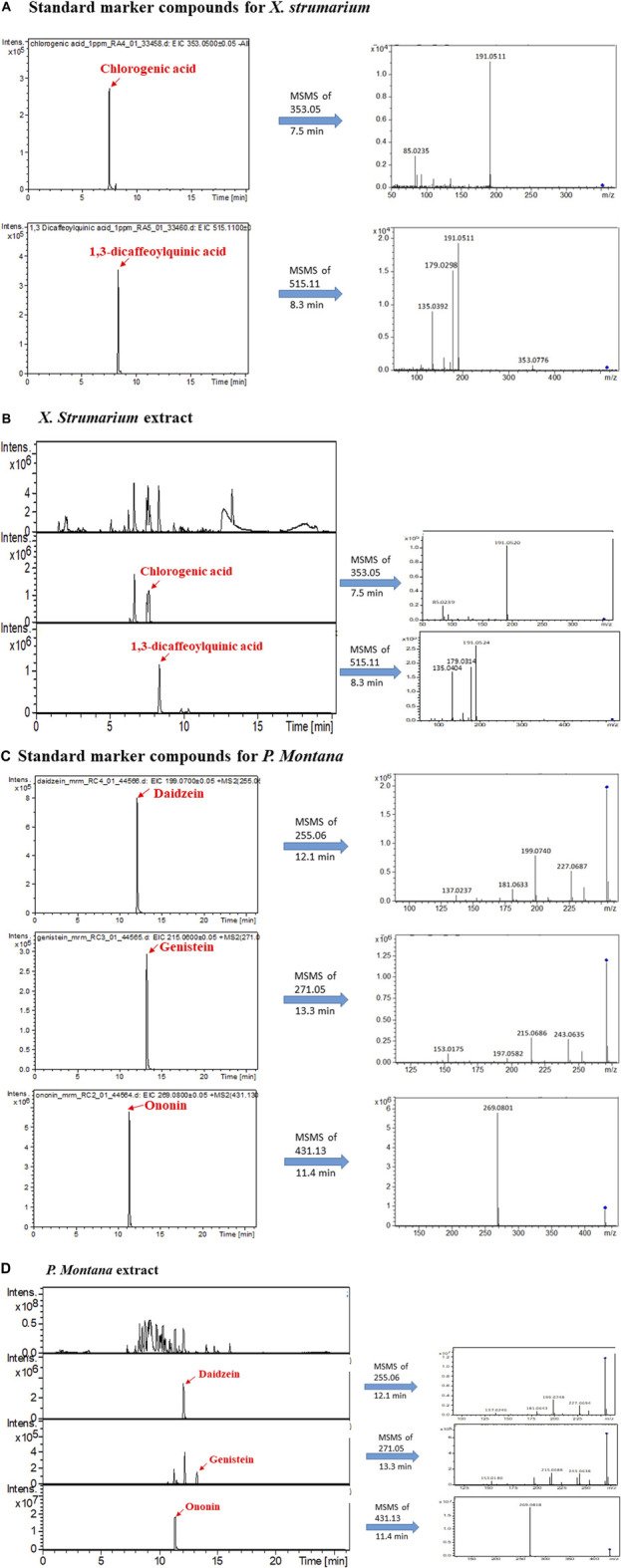
LC-MS/MS analysis of marker compounds and the extracts of *X. strumarium* and *P. montana*. **(A)** Extracted ion chromatogram (EIC) of standards of chlorogenic acid and 1,3-dicaffeoylquinic acid and their fragmented ions. **(B)** base peak chromatogram (BPC) and EIC of the *X. strumarium* extract. **(C)** EIC of standards of daidzein, genistein, and ononin and their fragmented ions. **(D)** BPC and EIC of the *P. montana* extract.

*X. strumarium and P. montana* related marker compounds (Figure 1) were obtained from commercial pharmaceutical or chemical companies. Chlorogenic acid (catalog number: CFN99116; purity: ≥98.0%), daidzein (catalog number: CFN98774; purity: ≥98.0%), genistein (catalog number: CFN98681; purity: ≥98.0%), and ononin (catalog number: CFN99136; purity: ≥98.0%) were purchased from ChemFaces Biochemical (Wuhan, Hubei, China). 1,3-dicaffeoylquinic acid (catalog number: D8196; purity: ≥98.0%) was purchased from Sigma-Aldrich, Inc. (St. Louis, MO, United States).

### Cell Viability Assay

J-Lat cell lines (clones 8.4 and 9.2) and Jurkat cells were cultured overnight in 96-well plates. Culture medium containing *P. montana* (1 mg/ml), *P. montana* (2 mg/ml), *X. strumarium* (1 mg/ml), *X. strumarium* (2 mg/ml), TNF-alpha (5 ng/ml), and TNF-alpha (10 ng/ml) were then added and incubated with cells for additional 24, 48, and 72 h at 37°C (Supplementary Figure S2). For *X. strumarium* related marker compounds, J-Lat cell lines (clones 8.4 and 9.2) were cultured overnight in 96-well plates. Culture medium containing chlorogenic acid (10 µM), chlorogenic acid (20 µM), chlorogenic acid (50 µM), chlorogenic acid (100 µM), 1,3-dicaffeoylquinic acid (10 µM), 1,3-dicaffeoylquinic acid (20 µM), 1,3-dicaffeoylquinic acid (50 µM), 1,3-dicaffeoylquinic acid (100 µM), and *X. strumarium* (2 mg/ml) were then added and incubated with cells for additional 48 h at 37°C (Supplementary Figure S3A–D). For prostratin, J-Lat cell lines (clones 8.4 and 9.2) were cultured overnight in 96-well plates. Culture medium containing prostratin (0.5 µM), prostratin (1 µM), and prostratin (5 µM) were then added and incubated with cells for an additional 48 h at 37°C (Supplementary Figure S3E, F).

This was followed by 4-[3-(4-iodophenyl)-2-(4-nitrophenyl)-2H-5-tetrazolio]-1,3-benzene disulfonate (WST-1) assay (Roche, Indianapolis) as described previously (Tsai et al., 2011; Lin Y.-J. et al., 2013). After treatment, 10 µL of WST-1 was added to each well, followed by incubation at 37°C for 1 h. Absorbance at 450 nm was measured against background controls using a 96-well plate reader. Cell survival rates were calculated as the ratio of the optical density of treated cells at 450 nm (OD450) to the OD450 of untreated cells. Four wells were analyzed for each concentration. The data shown in Supplementary Figures S2, S3 represent the mean ± SD for three independent experiments.

### Liquid Chromatography-Tandem Mass Spectrometry Analysis for Marker Compounds and the Extracts of *X. Strumarium* and *P. Montana*

A high-performance LC system (Ultimate 3000 LC; Dionex, Germany) coupled with a quadrupole-time-of-flight MS (Q-TOF MS) (maXis impact; Bruker, Taiwan Co. Ltd.) was used with the full scan, DDA and multiple reaction monitoring (MRM) function (Cheng et al., 2019).

For LC-MS analysis, an Atlantis T3 analytical column (C18, 3 μm, 2.1 × 150 mm; Waters, Milford, MA, United States) was used with a flow rate of 0.2 ml/min and the mobile phases of solvent A (0.1% formic acid) and solvent B (100% acetonitrile). Three LC gradient methods, A and B, were used to produce suitable separation conditions for different compounds. In method A, solvent B was maintained at 5% for 1 min and then increased to 99% for 18 min. After maintaining this for 2.5 min, solvent B was decreased to 5% and held at this concentration for 3 min at a flow rate of 0.2 ml/min. Method A was used to analyze the marker compounds of chlorogenic acid and 1,3-dicaffeoylquinic acid in *X. strumarium* (Figure 1). In method B, the flow rate was 0.25 ml/min, and solvent B was maintained at 1% for 3 min, then increased to 99% for 15 min. After maintaining this concentration for 4 min, solvent B was decreased to 1% and held at this concentration for 3 min. The B method was used to analyze the marker compounds of daidzein, genistein, and ononin in *P. montana* (Figure 1).

The mass spectrometer was operated in negative ion mode for chlorogenic acid and 1,3-dicaffeoylquinic acid analysis in the m/z range 50–1,000 at 2 Hz. The capillary voltage of the ion source was set at -2500 V for the negative mode, and the endplate offset was 500 V. The nebulizer gas flow was 1 bar, and the drying gas flow rate was 6 L/min. The drying temperature was set to 200°C. For daidzein, genistein, and ononin, the mass spectrometer was operated in positive ion mode using an m/z range of 50–800 at 2 Hz. The capillary voltage of the ion source was set at +4500 V, and the endplate offset was 500 V. The nebulizer gas flow was 1 bar, and the drying gas flow rate was 8 L/min. The drying temperature was set to 200°C.

### Western Blot Analysis

**FIGURE 2:**
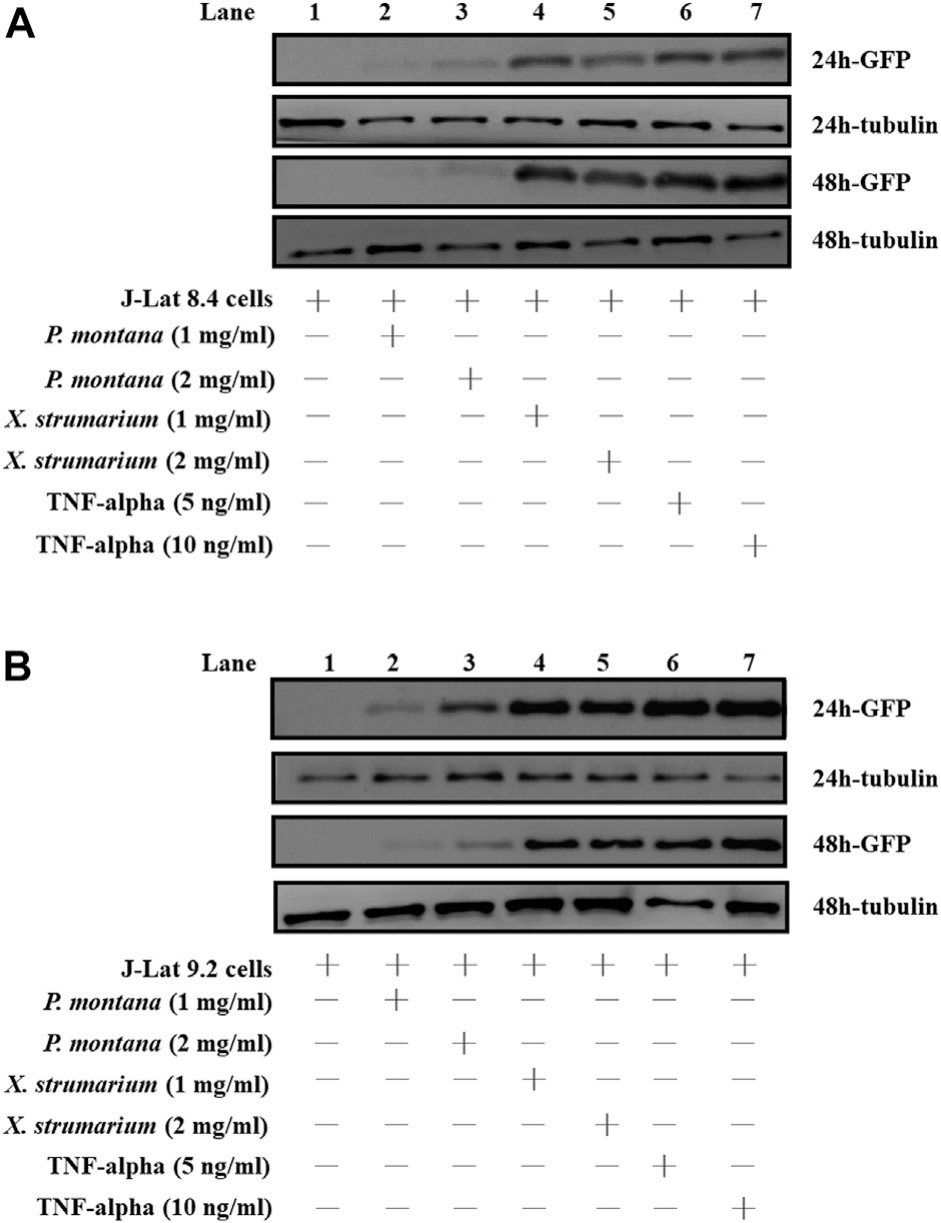
Effect of *P. montana* and *X. strumarium* on protein expressions under the control of HIV-1 5′- LTR in J-Lat cells, respectively. **(A)** J-Lat 8.4 cells and **(B)** J-Lat 9.2 cells were treated with herbal extracts for 24 and 48 h, respectively. J-Lat cells only were served as the negative controls (Lane 1). *P. montana* treated J-Lat cells were served as the experimental group (Land 2, *P. montana* (1 mg/ml); Lane 3, *P. montana* (2 mg/ml)). *X. strumarium* treated J-Lat cells were also served as the experimental group (Lane 4, *X. strumarium* (1 mg/ml); Lane 5, *X. strumarium* (2 mg/ml)). TNF-alpha (5 and 10 ng/ml) treated J-Lat cells were served as the positive controls (Lanes 6 and 7). The un-treated and treated cell lysates were then resolved by SDS-PAGE and Western blot analysis.

J-Lat cells were incubated with *X. strumarium and P. montana* (1 or 2 mg/ml) for 24 and 48 h, respectively, at 37°C, followed by Western blot analysis (Figures 2A,B). Cells treated with TNF-alpha (5 and 10 ng/ml) were used as positive controls. The cells were then lysed in RIPA buffer (catalog number 89900, Pierce, Thermo Fisher Scientific, Rockford, IL, United States) with a protease inhibitor (complete EDTA-free protease inhibitor, catalog number 11873580001, Roche Life Science, Sigma-Aldrich) and a phosphatase inhibitor (catalog number 88667, Pierce), subjected to 12% sodium dodecyl sulfate polyacrylamide gel electrophoresis, and then transferred to polyvinylidene fluoride membranes (Millipore, Billerica, MA, United States). The membranes were incubated with primary antibodies overnight at 4°C. The primary antibodies included anti-GFP (catalog number GTX113617), GeneTex, Inc. (Irvine, CA, United States), and anti-tubulin (catalog number 11224-1-AP) antibodies from Proteintech Group Inc. (Rosemont, IL, United States). Thereafter, the membranes were incubated with alkaline phosphatase-conjugated secondary antibodies (Sigma-Aldrich). Signals were visualized using a chemiluminescence kit (Chemicon), according to the manufacturer’s protocol.

### Enzyme-Linked Immunosorbent Assay

**FIGURE 3:**
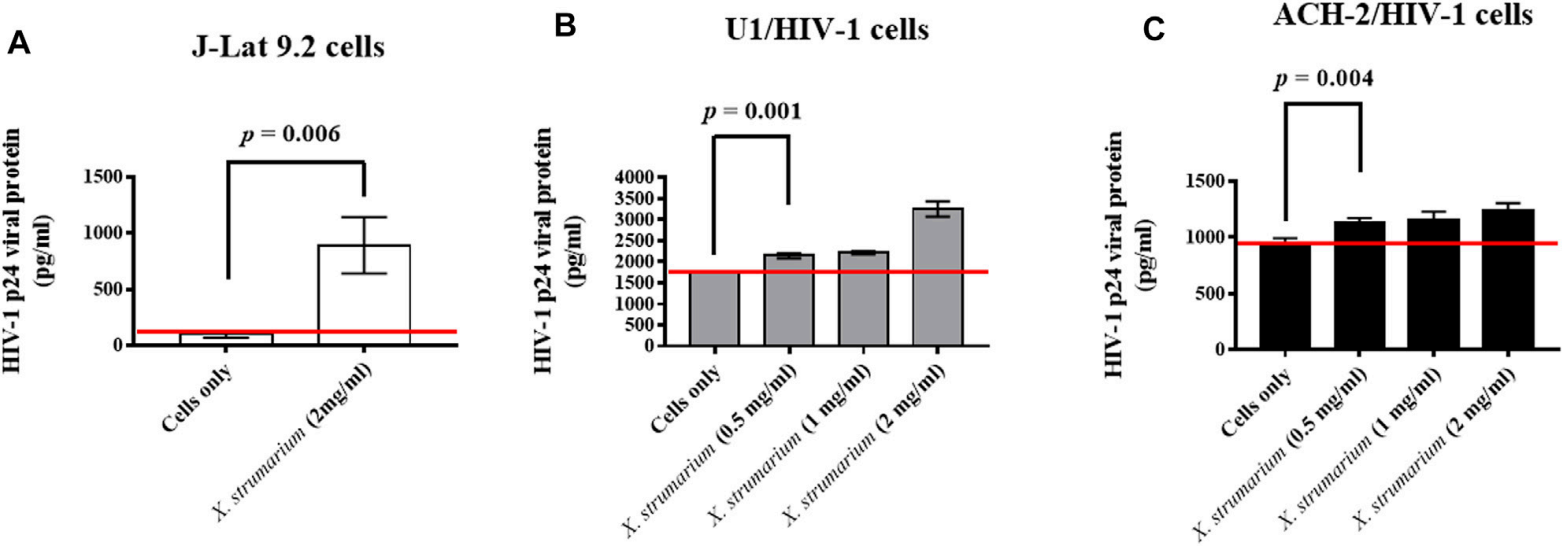
Effect of *X. strumarium* on HIV-1 p24 viral protein expressions under the control of HIV-1 5′- LTR in U1 and ACH-2 cells, respectively. **(A)** J-Lat 9.2 cells were treated with *X. strumarium* (2 mg/ml) for 48 h. **(B)** U1 cells were treated with *X. strumarium* within various concentrations (0, 0.5, 1, and 2 mg/ml) for 48 h. **(C)** ACH-2 cells were treated with *X. strumarium* within various concentrations (0, 0.5, 1, and 2 mg/ml) for 48 h. J-Lat 9.2, U1, or ACH-2 cells only were served as negative controls. The un-treated and treated cell culture supernatants were then detected by a HIV-1 p24 viral protein ELISA kit.

For J-Lat 9.2 cells, cells were incubated with *X. strumarium* (2 mg/ml) for 48 h (Figure 3A). For U1 cells, cells were incubated with *X. strumarium* (0.5, 1, and 2 mg/ml) for 48 h (Figure 3B). For ACH-2 cells, the cells were incubated with *X. strumarium* (0.5, 1, and 2 mg/ml) for 48 h (Figure 3C). Thereafter, the culture supernatants were collected and detected using a HIV p24 antigen enzyme-linked immunosorbent assay (ELISA) kit (Zeptometrix) according to the manufacturer’s instructions.

### Human Immunodeficiency Virus-1 5′-Long Terminal Repeat DNA-Affinity Extractions Using Capture Biotin-Tagged Probe Approach and Cell Nuclear Extracts

The pNL4-3 plasmid contained a HIV-1 5′- LTR fragment (corresponding to nt 1–789, where nt 1 was the start of the 5′-LTR U3 region) upstream of the *EGFP* gene (generously given by Prof. Chien-Hui Hung, Graduate Institute of Clinical Medical Sciences (Chiayi Branch) Chang-Gung University). The pNL4-3 plasmid was used as the DNA template for the *in vitro* synthesized 621-bp-long DNA fragment of the HIV-1 5′-LTR (GenBank AF324493.2: nt 1–621) by polymerase chain reaction (PCR). The following primers were used: forward primer, 5′-/desBioTEG/TGGAAGGGCTAATTTGGT-3′ and reverse primer: 5′-/Cy3/CCACACTGACTAAAAGGGTCTG-3′.

J-Lat 9.2 cells were treated with *X. strumarium* (2 mg/ml) for 24 h. The treated cells were harvested, washed, and resuspended in ice-cold PBS. Cells were used as negative controls. Thereafter, the treated cells and cell-only controls were lysed and extracted using a nuclear extraction kit (NE-PER Nuclear and Cytoplasmic Extraction Reagents, Thermo Scientific).

**FIGURE 4:**
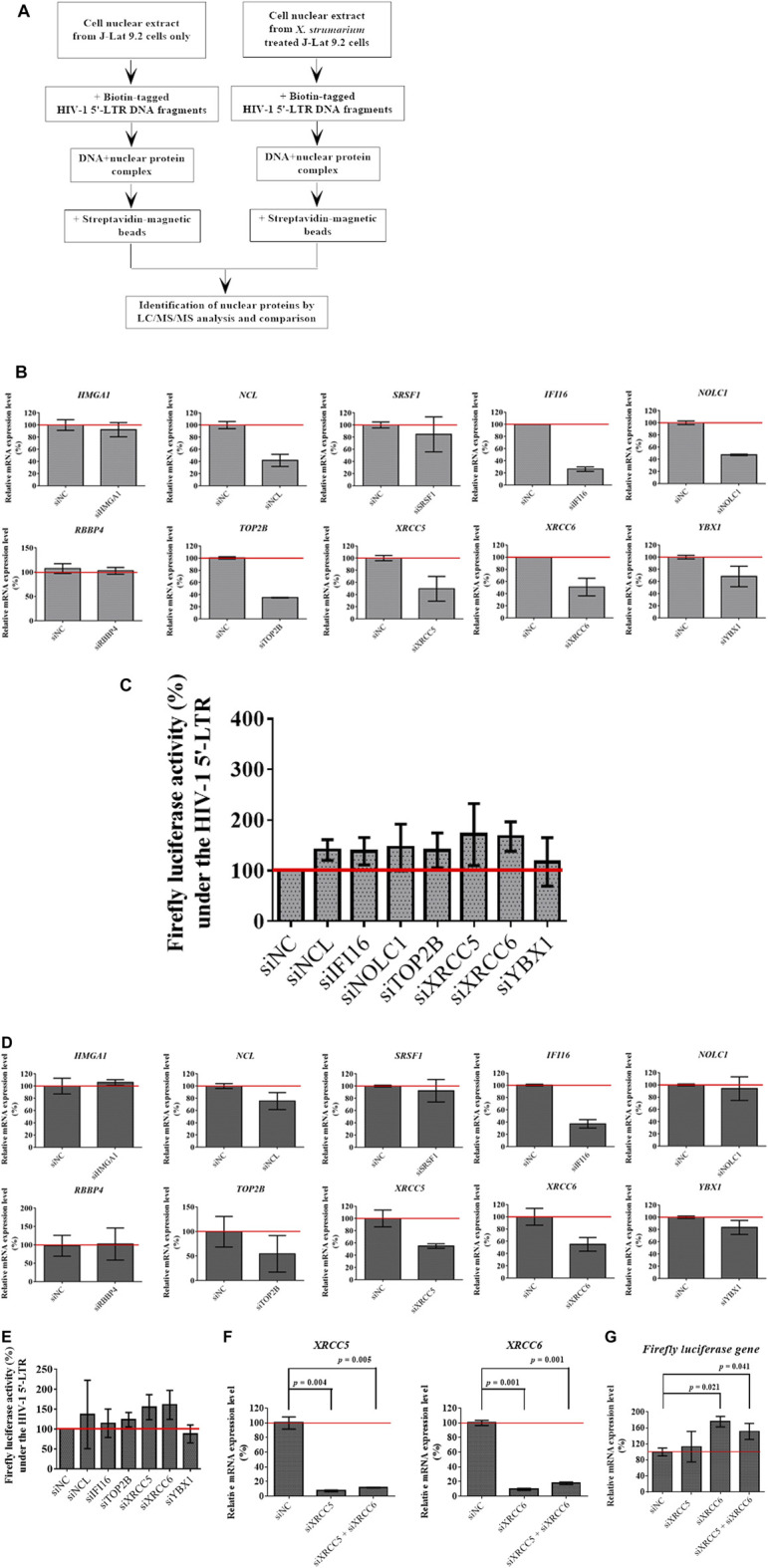
Identification of *X. strumarium*-associated nuclear regulatory proteins that were interacted with the HIV-1 5′- LTR region of the HIV-1 genome. **(A)** flow chart for DNA affinity capture of *X. strumarium*-associated nuclear regulatory proteins interacting with HIV-1 5′-LTR region (nt 229–455). **(B)** Relative mRNA expression level (%) of 10 nuclear regulatory genes- *HMGA1*, *NCL*, *SRSF1*, *IFI16*, *NOLC1*, *RBBP4*, *TOP2B*, *XRCC5*, *XRCC6*, and *YBX1* in TZM-bl cells with pcDNA HIV-1 viral tat-flag gene plasmid after transfection with siRNAs for these 10 interest genes, respectively for 24 h. The siRNAs of interest genes included siHMGA1, siNCL, siRBBP4, siYBX1, siUSF2, siIFI16, siNOLC1, siXRCC5, siXRCC6, siTOP2B, and siSRSF1. The scramble siRNAs were transfected into the TZM-bl cells and served as the negative controls (siNC). After 24 h transfection, the mRNA expression levels of these 10 interest genes were quantified by RT-qPCR method according to the manufacturer’s recommendations.

The J-Lat 9.2 cell nuclear extracts were pre-incubated with magnetic beads. Biotin-tagged HIV-1 5′-LTR DNA fragments were then incubated with J-Lat 9.2 cell nuclear extracts for 4 h at 4°C (Figure 4A). Biotin-tagged DNA/nuclear protein complexes were affinity-purified using streptavidin-magnetic beads. The biotin-tagged DNA/nuclear protein/streptavidin-magnetic bead complexes were washed with ice-cold PBS. Nuclear proteins bound to capture biotin-tagged probes were purified and prepared for LC-MS/MS analysis. All assays were performed in triplicate in three independent experiments.

### Liquid Chromatography-Tandem Mass Spectrometry Analysis for Protein Identification

The purified proteins were identified using a nanoflow UPLC system (UltiMate 3,000 RSLCnano system, Dionex, Netherlands) coupled to a quadrupole time-of-flight (Q-TOF) mass spectrometer (maXis impact, Bruker, Germany). The peptides were separated using an RP C18 capillary column (25 cm × 75 µm id) at a 300 nL/min flow rate, and eluted with a linear ACN gradient from 10 to 50% ACN in 0.1% formic acid for 90 min. The eluted peptides from the capillary column were sprayed into the MS using a captive spray ion source (Bruker, Germany). Data acquisition from Q-TOF was performed using Data Analysis software (version 4.1, Bruker, Germany). Proteins were identified by the nanoLC-MS/MS spectra (Table 1) by searching the SwissProt database using the MASCOT search algorithm (version 2.3.02).

**TABLE 1:** Nuclear regulatory proteins identified by HIV-1 5′-LTR DNA-affinity capture and LC-MS/MS analysis.

Protein name	Assession no	Gene name	Previously described interaction with HIV LTR	PMID no	Un-treated cells							*X. strumarium*-treated cells					
					Experiment 1		Experiment 2		Experiment 3		Experiment 1			Experiment 2		Experiment 3	
					**Mascot score**	**Peptide number**	**Mascot score**	**Peptide number**	**Mascot score**	**Peptide number**	**Mascot score**		**Peptide number**	**Mascot score**	**Peptide number**	**Mascot score**	**Peptide number**
HMGA1_HUMAN	P17096	*HMGA1*	Yes	23392246	57.3	2	0.0	0	58.6	1	100.6		3	97.1	2	54.4	1
ILF2_HUMAN	Q12905	*ILF2*	Yes	19454010, 22174317, 23125841	48.4	1	146.7	3	176.9	3	133.5		3	217.1	4	328.4	5
NUCL_HUMAN	P19338	*NCL*	Yes	19884766	0.0	0	0.0	0	174.2	4	29.3		1	69.6	2	212.6	3
SRSF1_HUMAN	Q07955	*SRSF1*	Yes	19716452	63.0	2	55.8	1	111.7	3	103.4		2	106.4	2	144.2	3
SRSF3_HUMAN	P84103	*SRSF3*	No		72.6	2	111.5	2	96.8	2	143.0		3	132.7	3	141.1	3
TCP4_HUMAN	P53999	*SUB1*	Yes	10887206	0.0	0	0.0	0	0.0	0	54.7		1	126.8	2	74.8	2
CAF1B_HUMAN	Q13112	*CHAF1B*	No		26.1	1	228.2	5	359.6	7	26.8		1	0.0	0	131.6	3
CBX3_HUMAN	Q13185	*CBX3*	Yes	23166591, 21875947	36.0	1	40.1	1	65.4	2	0.0		0	0.0	0	32.4	1
H2A1A_HUMAN	Q96QV6	*H2AC1*	Yes	14657027, 11689053, 11080476, 9566873	159.8	3	155.9	3	188.3	3	0.0		0	174.8	3	130.0	2
H33_HUMAN	P84243	*H3F3*	Yes	14657027	78.9	2	79.9	2	93.1	1	0.0		0	0.0	0	73.3	1
IF16_HUMAN	Q16666	*IFI16*	Yes	12539042	47.1	1	303.6	6	500.7	8	49.6		1	99.2	2	87.8	2
NFYB_HUMAN	P25208	*NFYB*	No		112.1	2	161.8	2	152.3	2	76.8		2	59.6	1	33.0	1
NFYC_HUMAN	Q13952	*NFYC*	No		98.3	2	187.5	2	286.2	4	50.5		1	142.6	2	113.9	2
NOLC1_HUMAN	Q14978	*NOLC1*	Yes	22190034	223.1	5	250.9	5	220.2	4	144.4		3	134.1	2	149.9	3
PSIP1_HUMAN	O75475	*PSIP1*	Yes	22190034	29.8	1	32.2	1	138.4	3	0.0		0	0.0	0	0.0	0
RBBP4_HUMAN	Q09028	*RBBP4*	Yes	19454010	366.5	7	333.9	5	237.4	5	292.3		6	270.0	5	180.9	3
RCC2_HUMAN	Q9P258	*RCC2*	Yes	22944692	102.3	3	41.4	1	209.8	5	28.6		1	52.8	1	124.8	3
TCF7_HUMAN	P36402	*TCF7*	No		132.6	2	171.4	2	98.4	2	0.0		0	0.0	0	0.0	0
TFAP4_HUMAN	Q01664	*TFAP4*	Yes	22077140, 18187620, 7933101	190.4	3	305.1	4	292.2	4	0.0		0	145.6	2	0.0	0
TFCP2_HUMAN	Q12800	*TFCP2*	Yes	11940654, 10888618, 9371597, 8289393	392.6	6	452.0	8	437.9	6	30.1		1	0.0	0	35.4	1
TOP2B_HUMAN	Q02880	*TOP2B*	Yes	23399433	169.5	4	86.1	2	367.9	8	0.0		0	0.0	0	0.0	0
UBIP1_HUMAN	Q9NZI7	*UBP1*	Yes	15207707, 7836461	271.8	4	132.4	2	167.3	4	0.0		0	0.0	0	0.0	0
USF1_HUMAN	P22415	*USF1*	Yes	8289399	259.8	3	347.3	4	198.2	2	265.3		3	170.5	2	155.0	2
USF2_HUMAN	Q15853	*USF2*	No		155.6	2	201.0	3	140.7	2	0.0		0	0.0	0	0.0	0
XRCC5_HUMAN	P13010	*XRCC5*	Yes	8240370	1,596.9	24	1897.1	28	1955.0	29	1,351.8		22	1,301.8	23	1,244.3	19
XRCC6_HUMAN	P12956	*XRCC6*	Yes	8240370	2058.8	29	2,206.8	30	2,621.4	35	1789.2		26	1798.1	25	2,175.4	29
YBOX1_HUMAN	P67809	*YBX1*	Yes	19454010, 10573156, 9472608, 8254772	125.7	2	39.3	1	88.0	1	95.3		2	54.1	1	0.0	0

### Luciferase Activity Under the Human Immunodeficiency Virus-1 5′-Long Terminal Repeat Control Coupled With siRNA Assay

The TZM-bl cells were transfected with pcDNA HIV-1 viral tat-flag gene plasmid and siRNAs including siHMGA1, siNCL, siRBBP4, siYBX1, siIFI16, siNOLC1, siXRCC5, siXRCC6, siTOP2B, and siSRSF1 for 24 h or 48 h, respectively (Supplementary Table S2, Figure 4B, and Figure 4D). siNC was used as a negative control. Scramble siRNAs (siNC) was transfected into TZM-bl cells. After 24 h or 48 h of incubation, the transfected cells were lysed using a cytoplasmic extraction kit (NE-PER Nuclear and Cytoplasmic Extraction Reagents, Thermo Scientific). Thereafter, the cell lysates were applied to detect firefly luciferase activity using the luciferase Assay System (Promega) according to the manufacturer’s recommendations. All assays were performed in triplicates in three independent experiments.

### Real-Time Polymerase Chain Reaction

Cellular RNA was isolated using a QIAamp® RNA Mini Kit according to the manufacturer’s instructions (Qiagen, Valencia, CA, United States). RNA was eluted in 60 μL buffer, and real-time TaqMan RT-PCR assays were used to determine siRNA knockdown efficiency. The primers used for quantitative PCR (qPCR) amplification were as follows: *HMGA1*, forward: 5′-GAAAAGGACGGCACTGAGAA-3′and reverse: 5′-CTC​TTA​GGT​GTT​GGC​ACT​TCG-3′; *NCL*, forward: 5′-CCA​CTT​GTC​CGC​TTC​ACA -3′and reverse: 5′-TCT​TGG​GGT​CAC​CTT​GAT​TT-3′; *SRSF1*, forward: 5′-GGCGGTCTGAAAACAGAGTG-3′and reverse: 5′-TTT​AAA​TCC​TGC​CAA​CTT​CCA-3′; *IFI16*, forward: 5′-ACT​CCT​CAG​ATG​CCA​CCA​AC-3′ and reverse: 5′-TCA​TTT​TGG​AGA​TTG​TGT​CTT​CAC-3′; *NOLC1*, forward: 5′-ATA​AGT​TCG​CCA​AAG​CGA​CA-3′ and reverse: 5′-CTA​AGA​GGG​AAG​AGG​CAT​TGG-3′; *RBBP4*, forward: 5′-CGG​ATG​AAC​AAA​ACC​ATC​TTG-3′ and reverse: 5′-CTG​AAC​CAA​AAC​CTC​CAA​ATT​C-3′; *TOP2B*, forward: 5′-AGC​CTG​GAA​AGA​AGC​ACA​AG-3′ and reverse: 5′-TCG​GAG​GAA​CTA​TCA​TCA​TGC-3′; *XRCC5*, forward: 5′- GAG​CCC​ACT​TCA​GCG​TCT-3′ and reverse: 5′-CAG​CAG​GAT​TCA​CAC​TTC​CA-3′; *XRCC6*, forward: 5′-CCT​TTT​GAC​ATG​AGC​ATC​CA-3′ and reverse: 5′-AAT​TTT​TGT​CTT​TCT​CGG​TAC​CAT-3′; *YBX1*, forward: 5′-GGA​GGG​TGC​TGA​CAA​CCA-3′ and reverse: 5′-GCT​GTC​TTT​GGC​GAG​GAG-3′; *firefly luciferase*, forward: 5′-AGG​TCT​TCC​CGA​CGA​TGA-3′ and reverse: 5′-GTC​TTT​CCG​TGC​TCC​AAA​AC-3′; *GFP*, forward: 5′-GAA​GCG​CGA​TCA​CAT​GGT-3′ and reverse: 5′-CCA​TGC​CGA​GAG​TGA​TCC-3′.

Reverse transcription was performed in a 10 μL reaction mixture consisting of 2 μL RNA template, 1 μL RT primer mix, 1 μL dNTP mix (10 mm each), and 6 μL RNA/DNAse-free water at 65°C for 5 min. Next, a reaction mixture of 4 μL 5 × MMLV buffer, 0.8 μL MMLV enzyme, and 5.2 μL RNA/DNAse-free water was added to each RNA sample. Reverse transcription reactions were performed at 42°C for 60 min cDNA was amplified by PCR in a 20 μL reaction mixture containing 5 μL cDNA, 10 μL, 2 × Mastermix, 1 μL primer/probe mix, and 4 μL RNA/DNAse-free water. Real-time TaqMan RT-PCR conditions were 95°C for 10 min, 50 cycles of 95°C for 10 s, and 60°C for 60 s. RNA levels were detected using a 7900HT Fast Real-Time PCR System (Life Technologies, Carlsbad, CA, United States).

### Statistical Analysis

A Student’s t-test was used to compare the differences between the two groups (Figure 3 and Figure 4). Statistical significance was set at *p* < 0.05. A statistical package (SPSS 19.0) was used for all analyses.

## Results

### Liquid Chromatography-Tandem Mass Spectrometry Analysis of *X. Strumarium* and *P. Montana*

To confirm the quality of *X. strumarium and P. montana*, their corresponding marker compounds (Mun and Mun, 2015; Yoo et al., 2015) were purchased and identified in their extracts using LC-MS/MS analysis. Figure 1A, B show that chlorogenic acid and 1,3-dicaffeoylquinic acid were identified in *X. strumarium* based on their retention time, precursor ions, and fragmented ions. Daidzein, genistein, and ononin were identified in the extracts of *P. montana* (Figure 1C, D).

### Effect of *X. Strumarium* on Protein Expressions Under the Control of Human Immunodeficiency Virus-1 5′-Long Terminal Repeat Region in J-Lat Cells

J-Lat cells included J-Lat 8.4 and 9.2 cells (Jordan et al., 2003). These J-Lat cells contained an *EGFP* gene under HIV-1 5′-long terminal repeat (LTR) control, allowing the detection of HIV-1 reactivation. In this study, to evaluate the HIV-1 reactivation activity for *X. strumarium and P. montana*, J-Lat cells were used (Figure 2). Water extracts (1 or 2 mg/ml) of *X. strumarium* or *P. montana* were incubated with J-Lat cells for 24 and 48 h, respectively. TNF-alpha (5 and 10 ng/ml)-treated cells served as the positive controls. The activation of EGFP protein expression under HIV-1 5′-LTR control was detected using SDS-PAGE and Western immunoblotting against anti-EGFP antibodies (Figure 2).

As shown, for J-Lat 8.4 cells, cells treated with 1 or 2 mg/ml *X. strumarium* (Lanes 4 and 5) resulted in significant EGFP protein expressions in the cell lysates at 24 and 48 h when compared with the cells only (Lane 1) (Figure 2A). TNF-alpha (5 and 10 ng/ml)-treated cells served as positive controls (Lanes 6 and 7). However, cells treated with 1 or 2 mg/ml *P. montana* (Lanes 2 and 3) resulted in very low EGFP protein expression when compared with the cells only (Lane 1) (Figure 2A). Tubulin is a housekeep gene (Greer et al., 2010) and is used as an internal control in this study. Similar reactivation results were also observed in J-Lat 9.2 cells (Figure 2B). Significant levels of EGFP expressions were also observed in *X. strumarium*-treated cells (Lanes 4 and 5). These results suggested that *X. strumarium* reactivated HIV-1 latently infected cells via inducing EGFP protein expressions under the HIV-1 5′-LTR control.

### Effect of *X. Strumarium* on Human Immunodeficiency Virus-1 Viral p24 Protein Expressions Under the Control of Human Immunodeficiency Virus-1 5′-Long Terminal Repeat Region in J-Lat 9.2, U1, and ACH-2 Cells

In this study, to evaluate the effect of *X. strumarium* on HIV-1 reactivation activity, HIV-1 p24 virus capsid proteins in the cell culture supernatants were detected in J-Lat 9.2, U1, and ACH-2 cells using enzyme-linked immunosorbent assay (ELISA) analysis (Figure 3). J-Lat 9.2 cells treated with *X. strumarium* (2 mg/ml) resulted in significantly increased HIV-1 p24 virus capsid protein expressions (900 pg/ml) in the cell culture supernatants at 48 h when compared with the un-treated cell controls (100 pg/ml) (Figure 3A).

The U1 cells (promonocyte-derived cell line) and ACH-2 cells (T cell-derived cell line) were the cells with the full-length HIV-1 proviruses to detect the reactivation of HIV-1 gene products (Emiliani et al., 1996; Emiliani et al., 1998). U1 cells treated with *X. strumarium* (0.5, 1, or 2 mg/ml) resulted in significant increased HIV-1 p24 virus capsid protein expressions (2,142, 2,215, and 3,252 pg/ml, respectively; *p* = 0.001 for *X. strumarium* (0.5 mg/ml)) in the cell culture supernatants at 48 h when compared with the un-treated cell controls (1765 pg/ml) (Figure 3B). ACH-2 cells treated with *X. strumarium* (0.5, 1, or 2 mg/ml) also resulted in increased HIV-1 p24 virus capsid protein expressions (1,135, 1,150, and 1,238 pg/ml, respectively; *p* = 0.004 for *X. strumarium* (0.5 mg/ml)) at 48 h when compared with the un-treated cell controls (950 pg/ml) (Figure 3C). These results suggested that *X. strumarium* reactivated J-Lat 9.2 cells and another two kinds of HIV-1 latently infected cells by inducing HIV-1 p24 virus capsid protein expression under the HIV-1 5′-LTR control.

### Identification of *X. Strumarium*-Associated Nuclear Regulatory Proteins that Were Interacted With the Human Immunodeficiency Virus-1 5′- Long Terminal Repeat Region of the Human Immunodeficiency Virus Type 1 Genome

Our results showed that *X. strumarium* exhibited HIV-1 reactivation activity, which was controlled by HIV-1 5′-LTR. HIV-1 5′- LTR-associated nuclear regulatory proteins may be involved in HIV-1 reactivation. To identify nuclear regulatory proteins affected by *X. strumarium*, J-Lat 9.2 cells were treated with or without *X. strumarium*. The cell nuclear extracts then interacted with the biotin-labeled HIV-1 5′- LTR DNA fragments (Figure 4A). The streptavidin-magnetic bead pull-down assay was then used to capture the nuclear regulatory proteins associated with HIV-1 5′- LTR. LC-MS/MS analysis was then applied to identify nuclear regulatory proteins.

The nuclear regulatory proteins identified are listed in Table 1. For the increased nuclear regulatory proteins after *X. strumarium* treatment, there were 27 nuclear regulatory proteins associated with the HIV-1 5′-LTR region (Table 1). These included HMGA1, ILF2, NCL, SRSF1, SRSF3, SUB1, CHAF1B, CBX3, H2AC1, H3F3, IFI16, NFYB, NFYC, NOLC1, PSIP1, RBBP4, RCC2, TCF7, TFAP4, TFCP2, TOP2B, UBP1, USF1, USF2, XRCC5, XRCC6, and YBX1. Thereafter, these 27 nuclear regulatory proteins were subjected to Ingenuity Upstream Regulator Analysis using Ingenuity Pathway Analysis (IPA) to identify potential mechanistic upstream regulator signal transduction. As shown in Supplementary Table S1, there were 10 of the 27 nuclear regulatory proteins associated with upstream regulators, including MAX, MYC, NFKBIA, and E2F1. These 10 nuclear regulatory proteins included HMGA1, NCL, SRSF1, IFI16, NOLC1, RBBP4, TOP2B, XRCC5, XRCC6, and YBX1.

To investigate the role of these 10 identified nuclear regulatory proteins in HIV-1 reactivation activity under the control of the HIV-1 5′-LTR region, the TZM-bl cells transfected with pcDNA HIV-1 viral tat-flag gene plasmid and siRNAs for the 10 genes of interest were used in this study after 24 and 48 h transfection, respectively (Figures 4B,D). As shown in Figure 4B, after 24 h of transfection, silencing of *NCL*, *IFI16*, *NOLC1*, *TOP2B*, *XRCC5*, *XRCC6*, and *YBX1*, respectively, effectively inhibited their mRNA expression levels when compared with the siNC controls (siNC: 100%; siNCL: 40%; siIFI16: 25%; siNOLC1: 50%; siTOP2B: 35%; siXRCC5: 50%; siXRCC6: 50%; and siYBX1: 70%). There were no significant differences in *HMGA1*, *SRSF1*, and *RBBP4* mRNA expression between siRNA-treated and siNC-treated cells (Figure 4B). Cells transfected with siNCL, siIFI16, siNOLC1, siTOP2B, siXRCC5, siXRCC6, and siYBX1 were then detected for firefly luciferase activity at 24 h after transfection (Figure 4C). As shown in Figure 4C, cells transfected with siNCL, siIFI16, siXRCC5, and siXRCC6, respectively, resulted in increased luciferase activity at 24 h under the control of HIV-1 5′-LTR region when compared with the siNC transfected cells (siNC: 100%; siNCL: 140%; siIFI16: 140%; siXRCC5: 170%; and siXRCC6: 170%).

As shown in Figure 4D, after 48 h transfection, silencing of *NCL*, *IFI16*, *TOP2B*, *XRCC5*, *XRCC6*, and *YBX1*, respectively, effectively inhibited their mRNA expression levels when compared with the siNC controls (siNC: 100%; siNCL: 75%; siIFI16: 40%; siTOP2B: 50%; siXRCC5: 50%; siXRCC6: 55%; and siYBX1: 80%). There were no significant differences in *HMGA1*, *SRSF1*, *NOLC1*, and *RBBP4* mRNA expression between siRNA-treated and siNC-treated cells (Figure 4D). Cells transfected with siNCL, siIFI16, siTOP2B, siXRCC5, siXRCC6, and siYBX1 were then detected for firefly luciferase activity at 48 h after transfection (Figure 4E). As shown in Figure 4E, cells transfected with siXRCC5 and siXRCC6, respectively, showed increased luciferase activity at 48 h under the control of the HIV-1 5′-LTR region when compared with the siNC transfected cells (siNC: 100%; siXRCC5: 155%; and siXRCC6: 160%). The 24 and 48 h siRNA transfection results indicated that silencing of *XRCC5* and *XRCC6* resulted in increased luciferase activity, suggesting that XRCC5 and XRCC6 may play roles in the reactivation activity under the HIV-1 5′-LTR control.

To investigate the effect of *XRCC5* and *XRCC6* in mRNA level on HIV-1 reactivation activity in the HIV-1 5′-LTR region, TZM-bl cells transfected with pcDNA HIV-1 viral tat-flag gene plasmid and siXRCC5, siXRCC6, and siXRCC5 + siXRCC6 were used after 48 h transfection (Figures 4F,G). As shown in Figure 4F, after 48 h transfection, silencing (siRNAs) of *XRCC5* and *XRCC6*, respectively, effectively inhibited their mRNA expression levels when compared with the siNC controls (siNC: 100%; siXRCC5: 10%; and siXRCC6: 10%). Cells transfected with siXRCC6 and siXRCC5 + siXRCC6, respectively, resulted in increased mRNA levels of the firefly luciferase gene when compared with the siNC transfected cells at 48 h after transfection (siNC: 100%; siXRCC6: 175%; and siXRCC5 + siXRCC6: 150%; Figure 4G) (*p* = 0.021 for siXRCC6; *p* = 0.041 for siXRCC5 + XRCC6).

To investigate the relationship between *X. strumarium* and the 2 nuclear regulatory proteins- XRCC5 and XRCC6, J-Lat 8.4 cells were treated with *X. strumarium* (2 mg/ml) for 48 h (Supplementary Figure S1). The mRNA levels of *GFP*, *XRCC5*, and *XRCC6* were quantified by RT-qPCR. As shown in Supplementary Figure S1A, after 48 h treatment, *X. strumarium* (2 mg/ml) reactivated the *GFP* mRNA expressions to 35,000% when compared with the untreated cells (J-Lat 8.4 cells only: 100%). As shown in Supplementary Figure S1B, after 48 h of treatment, there were no significant differences in *XRCC5* mRNA expression between the untreated and treated cells. As shown in Supplementary Figure S1C, after 48 h of treatment, *XRCC6* mRNA expression was reduced to 10% in the *X. strumarium* (2 mg/ml)-treated cells when compared with the untreated cells (J-Lat 8.4 cells only: 100%). These results suggest that *X. strumarium* may reduce *XRCC6* mRNA expression. Silencing of 5′-LTR associated XRCC6 nuclear regulatory protein may reactivate latent HIV-1 infected cells *in vitro*.

## Discussion

Chinese herbal medicines (CHMs) exhibit multiple pharmacological properties. Chinese herbal medicines (CHMs) have been widely used in Taiwan, including in HIV-1 infected patients (Tsai et al., 2018). This study found that *X. strumarium* reactivated three HIV-1 latent cell models. Our deoxyribonucleic acid (DNA) affinity pull-down assay identified 27 nuclear regulatory proteins were associated with HIV-1 5′-LTR region in *X. strumarium* treated cells. In addition, silencing of the *XRCC6* nuclear regulatory protein reactivated HIV-1 5′-LTR promoter activity.

We first found that *X. strumarium* reactivated the HIV-1 5′-LTR driven transcription of the GFP reporter gene in J-Lat 8.4 and J-Lat 9.2 cells. In addition, *X. strumarium* exhibited the activation activity of the HIV-1 5′-LTR-driven protein expressions in J-Lat 9.2, U1, and ACH-2 cells by inducing HIV-1 viral p24 protein expressions. No similar studies have reported that *X. strumarium* can reactivate HIV-1 latently infected cells. Cary et al. showed that *Euphorbia kansui,* a CHM, can effectively activate CD4^+^ T cells and reactivate HIV-1 from latency, particularly when combined with HDACi (SAHA) or BETi (JQ1) (Cary et al., 2016). EK-16A, an ingenol derivative isolated from *Euphorbia kansui*, has been demonstrated to be 200-fold more potent than prostratin in reactivating latent HIV-1. Some natural compounds can be used with prostratin to activate HIV-1 5′-LTR, such as calcineurin and quercetin. Calcineurin enhances the non-tumor-promoting nuclear factor-kappaB (NF-κB) inducer prostratin and stimulates the latent HIV reservoir (Chan et al., 2013). A combination of quercetin and prostratin reactivates latent HIV-1 gene expression (Yang et al., 2018). This study observed that *X. strumarium* reactivated HIV-1 latently infected cells *in vitro*. Further studies in isolating active compounds from *X. strumarium* should be performed.

Our results showed that deoxyribonucleic acid (DNA) affinity pull-down assay identified 27 nuclear regulatory proteins associated with the HIV-1 5′-LTR region from the *X. strumarium*-treated nuclear extracts. We also found that *X. strumarium* may reduce *XRCC6* expressions in the mRNA level (Supplementary Figure S1). The reactivation activity by silencing X-ray repair cross-complementing 6 (XRCC6) reactivated protein expression under the control of the HIV-1 5′-LTR region. The HIV-1 5′-LTR contains many binding sites for cellular transcription factors for transcriptional regulation (Perkins et al., 1993; Perkins et al., 1994; El Kharroubi et al., 1998; Scalabrin et al., 2017; Wang et al., 2019; Ait Ammar et al., 2021). These HIV-1 5′-LTR interacting protein observations may offer therapeutic approaches for triggering the switch from latency to active replication, thereby eliminating HIV-1 latent infection (Vemula et al., 2015).

Our study showed that the reactivation activity of the HIV-1 5′-LTR region was silenced by X-ray repair cross-complementing 6 (*XRCC6*). Ku is a dimeric protein complex that binds to DNA double-strand break ends and is required for the non-homologous end joining (NHEJ) pathway of DNA repair. The Ku heterodimer acts as a transcription repressor and regulates transcription from the HIV-1 promoter (Jeanson and Mouscadet, 2002; Shadrina et al., 2016). Ku70 (X-ray repair cross-complementing 6, XRCC6) and Ku80 (X-ray repair cross-complementing 5, XRCC5) are two subunits of human Ku proteins encoded by the *XRCC6* and *XRCC5* genes, respectively. The XRCC5 and XRCC6 dimers act as regulatory factors of DNA-dependent protein kinase (DNA-PK) by increasing the affinity of the catalytic subunit PRKDC to DNA by 100-fold. The XRCC5 and XRCC6 dimers are involved in stabilizing the broken DNA ends and bringing them together. The XRCC5 and XRCC6 dimers, together with APEX1, act as negative regulators of transcription.

To our knowledge, this study is the first to demonstrate that *X. strumarium* may modulate the composition of these 5′-LTR associated nuclear regulatory proteins, affect 5′-LTR promoter activity, and reactivate HIV-1 latently infected cells *in vitro*. We identified 27 nuclear regulatory proteins associated with the HIV-1 5′-LTR region in *X. strumarium*-treated cells. Silencing of *XRCC6* reactivates HIV-1 5′-LTR transcriptional activity. A limitation of this study was the lack of the use of primary human cells and isolation of active compounds from *X. strumarium*. However, with the use of current cell lines, we observed that *X. strumarium* reactivated HIV-1 *in vitro*. Future studies on primary human cells and isolation of active compounds from *X. strumarium* will be conducted. These findings may contribute to understanding the viral 5′-LTR promoter activity and the mechanism by which drug-induced host cell nuclear regulatory proteins reactivate HIV-1 and for future investigations to eradicate and cure HIV-1 infection.

## Data Availability

The raw data supporting the conclusions of this article will be made available by the authors, without undue reservation.

## Abbreviations

HIV-1, human immunodeficiency virus type 1; HAART, highly active anti-retroviral therapy; AIDS, acquired immunodeficiency syndrome; LRA, Latency reversal agents; CHM, Chinese herbal medicine; ELISA, enzyme-linked immunosorbent assay; LC-MS/MS, liquid chromatography-tandem mass spectrometry; EGFP, enhanced green fluorescent protein; DNA, deoxyribonucleic acid; XRCC5, X-ray repair cross complementing 5; XRCC6, X-ray repair cross complementing 6; HDAC, Histone deacetylases; DNMT, DNA methyltransferase; TAR, tat responsive element; FBS, fetal bovine serum; CD4, cluster of differentiation 4 receptors; CCR5 C-C, chemokine receptor type 5; GMP, good manufacturing process.
