# Supplementary material for: Effect of Xanthium Strumarium on HIV-1 5′-LTR Transcriptional Activity and Viral Reactivation in Latently Infected Cells
Source: Front Pharmacol. 2021 Aug 6;12:720821. doi: 10.3389/fphar.2021.720821 (PMC8378250; doi:10.3389/fphar.2021.720821)
Supplement: Supplementary file 1 [file DataSheet1.docx]

**Supplementary Material**

**Supplementary Text**

**Figure S1**

**Figure S2**

**Figure S3**

**Figure S4**

**Table S1**

**Table S2**

**Supplementary Text**

**Figure S1 | Relative mRNA expression level (%) of *GFP*, *XRCC5*, and *XRCC6* in J-Lat 8.4 cells treated with *X. strumarium* for 48 h**. J-Lat 8.4 cells were treated with *X. strumarium* (2 mg/ml) for 48 h. J-Lat 8.4 cells only were served as the negative controls. After 48 h treatment, the mRNA expression levels of *GFP*, *XRCC5*, and *XRCC6* were quantified by RT-qPCR method.

**Figure S2 | Relative cell survival rate (%) of *P. montana*, *X. strumarium*, and TNF-alpha treated cells. J-Lat 8.4 cells, (B) J-Lat 9.2 cells, and (C) Jurkat cells.** Cells were treated with herbal extracts for 24 h, 48 h and 72 h, respectively. J-Lat or Jurkat cells only were served as the negative controls. *P. montana* treated cells were served as the experimental group (*P. montana* (1 mg/ml); *P. montana* (2 mg/ml)). *X. strumarium* treated cells were also served as the experimental group (*X. strumarium* (1 mg/ml); *X. strumarium* (2 mg/ml)). TNF-alpha (5 and 10 ng/ml) treated cells were served as the positive controls. The un-treated and treated cell lysates were detected using WST-1 assays. The percentage of cell survival rate was calculated as (OD450_cell+drug_)/(OD450_cell_) x 100%. Four wells were analyzed for each concentration.

**Figure S3 | Relative cell survival rate (%) of marker compounds (chlorogenic acid and 1,3-dicaffeoylquinic acid), *X. strumarium*, and prostratin treated cells. (A) chlorogenic acid in J-Lat 8.4 cells, (B) 1,3-dicaffeoylquinic acid in J-Lat 8.4 cells, (C) chlorogenic acid in J-Lat 9.2 cells, (D) 1,3-dicaffeoylquinic acid in J-Lat 9.2 cells, (E) prostratin in J-Lat 8.4 cells, and (F) prostratin in J-Lat 9.2 cells.** Cells were treated with marker compounds, *X. strumarium*, and prostratin for 48 h, respectively. J-Lat cells only were served as the negative controls. *X. strumarium* and prostratin treated cells were served as the positive controls. The un-treated and treated cell lysates were detected using WST-1 assays. The percentage of cell survival rate was calculated as (OD450_cell+drug_)/(OD450_cell_) x 100%. Four wells were analyzed for each concentration.

**Figure S4 | Effect of marker compounds (chlorogenic acid and 1,3-dicaffeoylquinic acid) on protein expressions under the control of HIV-1 5′- LTR in J-Lat cells, respectively.** (**A**) **J-Lat 8.4 cells** and **(B) J-Lat 9.2 cells.** Cells were treated with marker compounds for 48 h, respectively. J-Lat cells only were served as the negative controls (Lane 1). Marker compounds (chlorogenic acid and 1,3-dicaffeoylquinic acid) treated J-Lat cells were served as the experimental group (Land 3, chlorogenic acid (50 µM); Lane 4, 1,3-dicaffeoylquinic acid (20 µM)). *X. strumarium* (2 mg/ml) treated J-Lat cells were served as the positive controls (Lane 2). Prostratin (5 µM) treated J-Lat cells were also served as the positive controls (Lane 5). The un-treated and treated cell lysates were then resolved by SDS-PAGE and Western blot analysis.

**Table S1 |** Ingenuity Upstream Regulator Analysis using Ingenuity Pathway Analysis (IPA) to identify potential mechanistic upstream regulator signal transduction. Ingenuity pathway analysis (IPA) identified 10 proteins for *X. strumarium* -associated nuclear regulatory proteins from the LC-MS/MS analysis. There 10 proteins included HMGA1, NCL, RBBP4, YBX1, IFI16, NOLC1, XRCC5, XRCC6, TOP2B, and SRSF1. These 10 proteins were regulated by the 4 up-stream regulators- MAX, MYC, NFKBIA, and E2F1.

**Table S2 |** siRNA sequences in this study.

**Fig. S1.**


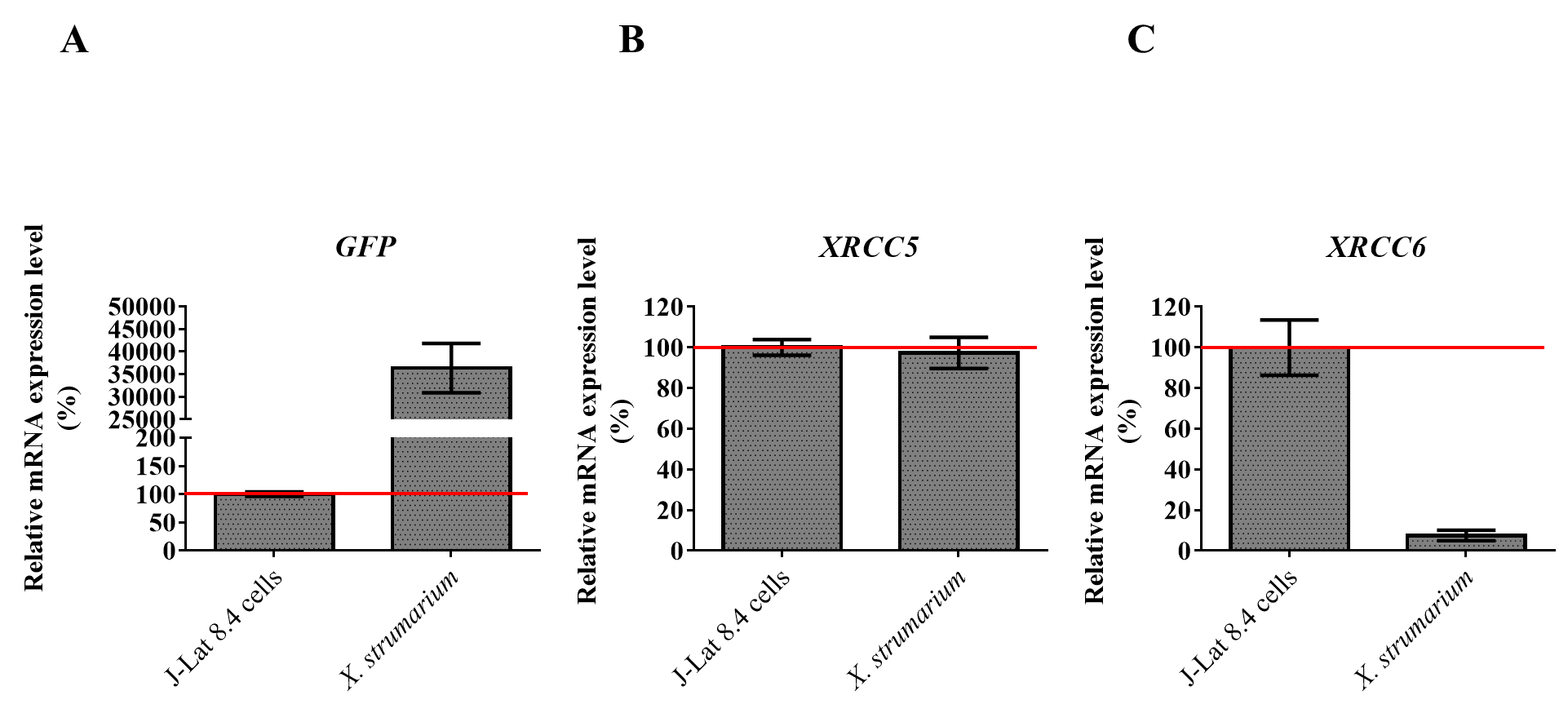


**Fig. S2.**


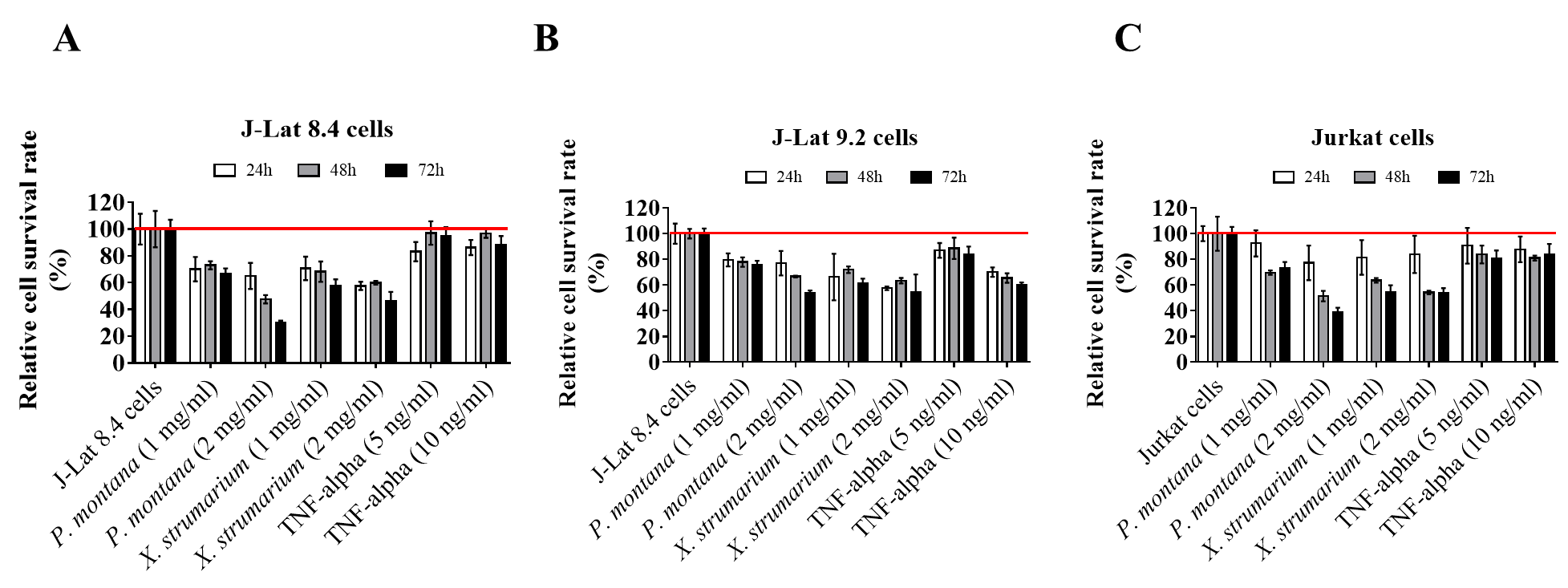


**Fig. S3.**


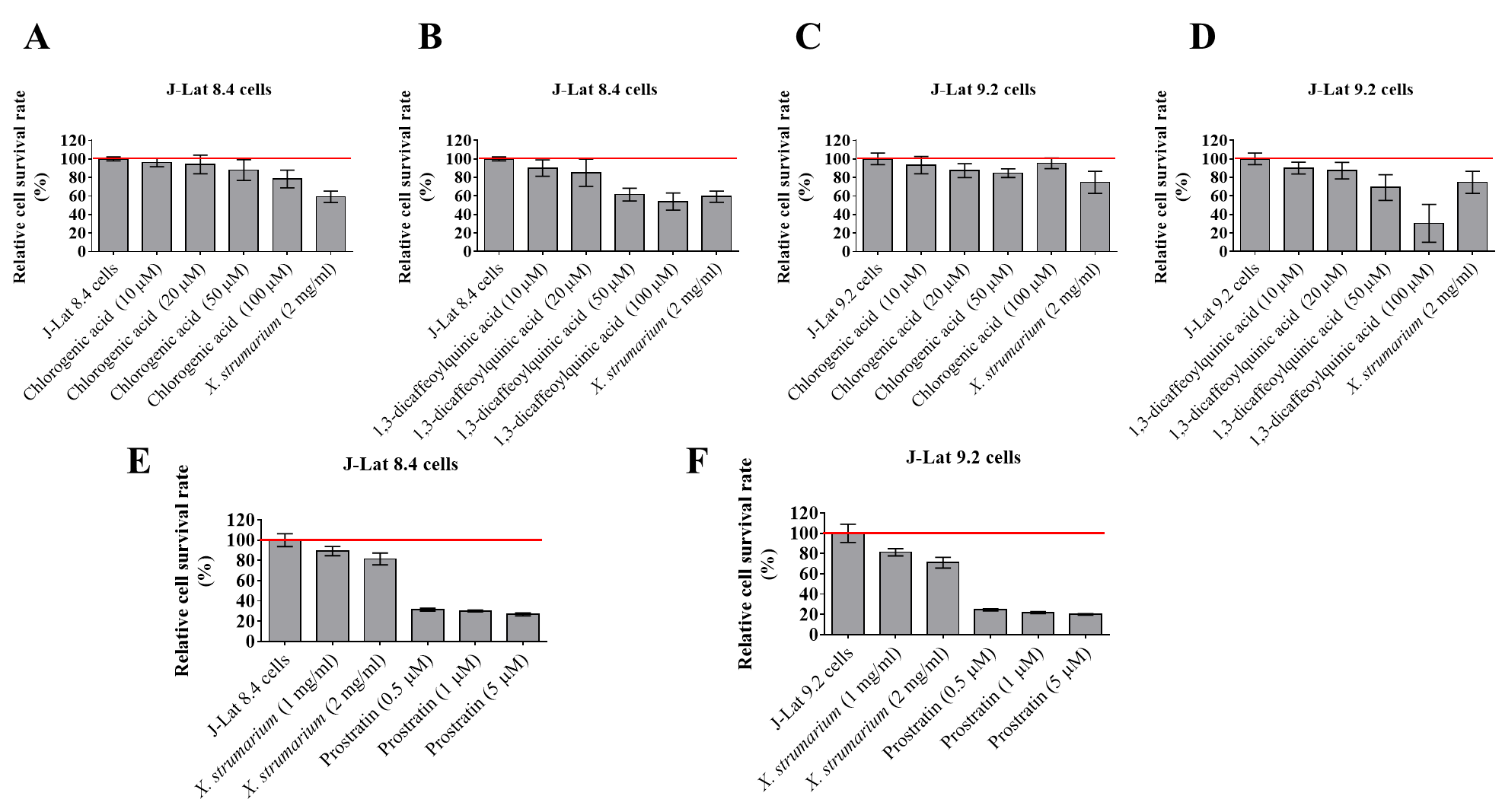


**Fig. S4.**


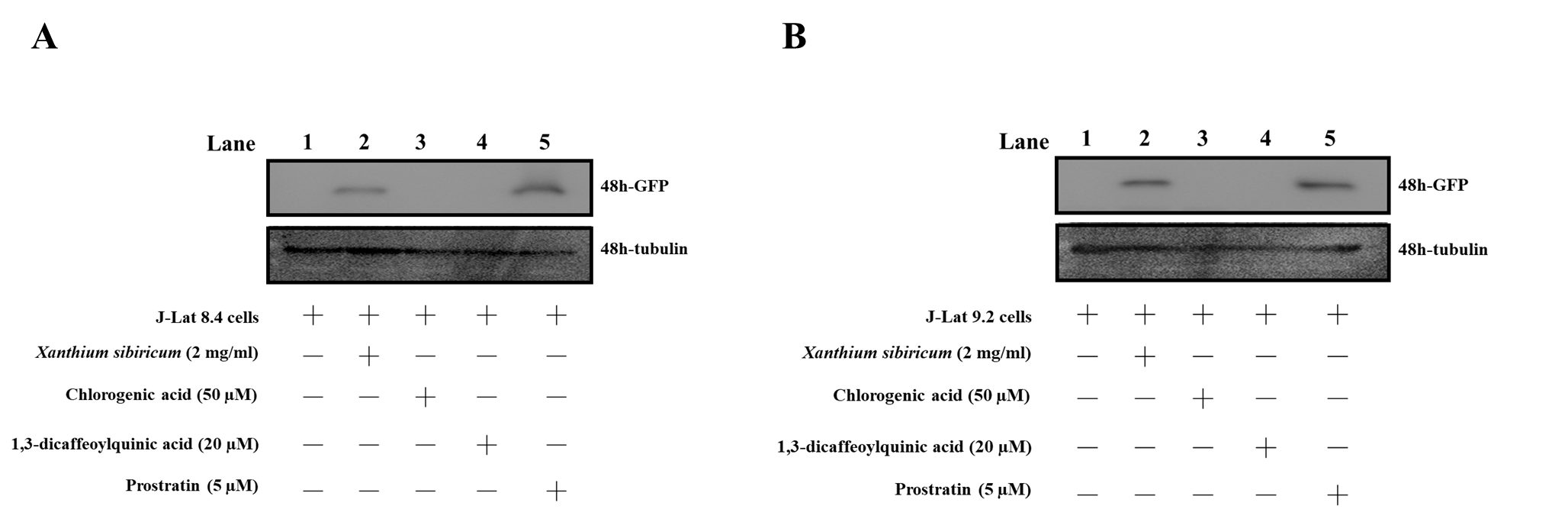


**Table S1 |** Ingenuity Upstream Regulator Analysis using Ingenuity Pathway Analysis (IPA) to identify potential mechanistic upstream regulator signal transduction. Ingenuity pathway analysis (IPA) identified 10 proteins for *X. strumarium* -associated nuclear regulatory proteins from the LC-MS/MS analysis. There 10 proteins included HMGA1, NCL, RBBP4, YBX1, IFI16, NOLC1, XRCC5, XRCC6, TOP2B, and SRSF1. These 10 proteins were regulated by the 4 up-stream regulators- MAX, MYC, NFKBIA, and E2F1.

**
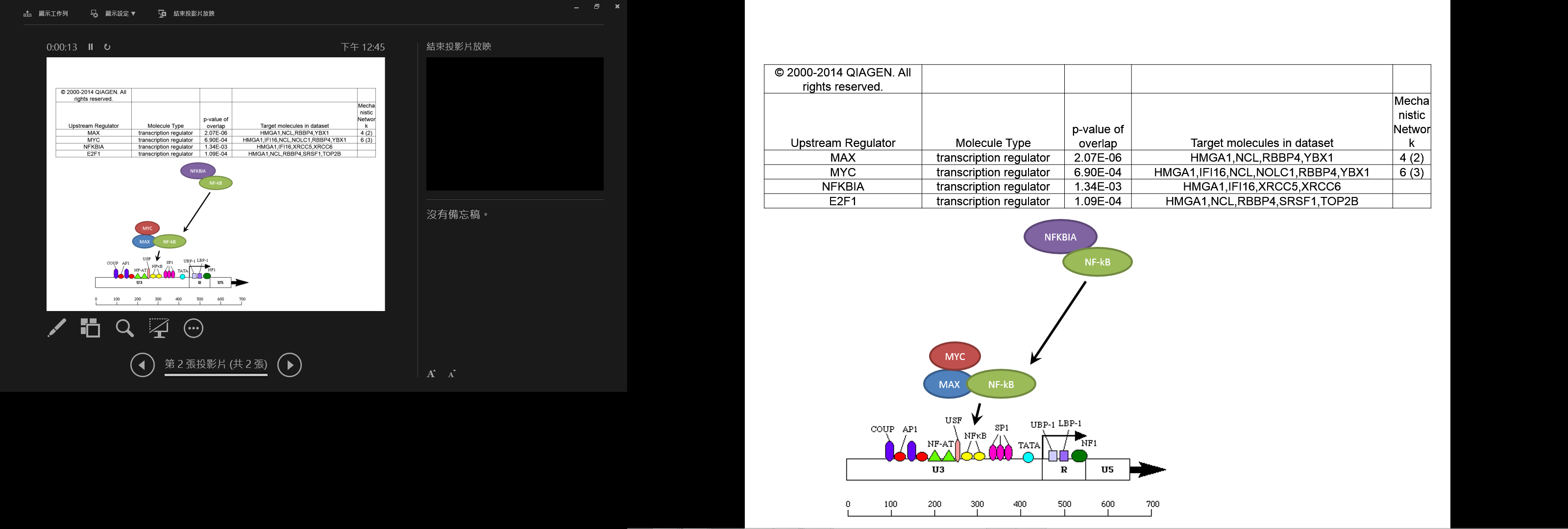
**

| **Table S2 \|** siRNA sequences in this study. | | | | |
| --- | --- | --- | --- | --- |
| siRNA | ON-TARGETplus SMARTpool (Dharmacon) | | | |
|  | duplex 1 | duplex 2 | duplex 3 | duplex 4 |
| siHMGA1 | GCGAAGUGCCAACACCUAA | GAGGGCAUCUCGCAGGAGU | CACCACAACUCCAGGAAGG | GAGGAGCAGUGACCCAUGC |
| siNCL | GCAAAGAAGGUGGUCGUUU | GAUAGUUACUGACCGGGAA | CAAAUCUGCUCCUGAAUUA | GAAAGAAGACGAAGUUUGA |
| siSRSF1 | GAAAGAAGAUAUGACCUAU | UAACUUACCUCCAGACAUC | UGAAGCAGGUGAUGUAUGU | CGACGGCUAUGAUUACGAU |
| siIFI16 | CAGCGUAACUCCUAAAAUC | GGAGUAAGGUGUCCGAGGA | GGACCAGCCCUAUCAAGAA | GAUCUGUAAUUCAUAGUCA |
| siNOLC1 | CAUCUAAGUCUGCAGUUAA | GGUCCCAGAGCGAAAGUUA | UGAGGUGGAUUCACGAGUU | GUCCAGGUCAAUUCUAUUA |
| siRBBP4 | GAUACUCGUUCAAACAAUA | GCAUACGGCAGUAGUAGAA | GCUAUGGGCUUUCUUGGAA | GACUGAAUGUCUGGGAUUU |
| siTOP2B | GAAGUUGUCUGUUGAGAGA | CGAAAGACCUAAAUACACA | GAUCAUAUGGGAUGUCUGA | GGUGUAUGAUGAAGAUGUA |
| siXRCC5 | GCAUGGAUGUGAUUCAACA | CGAGUAACCAGCUCAUAAA | GAGCAGCGCUUUAACAACU | AAACUUCCGUGUUCUAGUG |
| siXRCC6 | AUAAAGCUCUAUCGGGAAA | CAGGGUGGGAGUCAUAUUA | UUAGUGAUGUCCAAUUCAA | GAUCCAGGUUUGAUGCUCA |
| siYBX1 | CUGAGUAAAUGCCGGCUUA | CGACGCAGACGCCCAGAAA | GUAAGGAACGGAUAUGGUU | GCGGAGGCAGCAAAUGUUA |
| siNC | UGGUUUACAUGUCGACUAA | UGGUUUACAUGUUGUGUGA | UGGUUUACAUGUUUUCUGA | UGGUUUACAUGUUUUCCUA |
